# Multidrug-Resistant *Escherichia coli* Causing Urinary Tract Infections among Controlled and Uncontrolled Type 2 Diabetic Patients at Laquintinie Hospital in Douala, Cameroon

**DOI:** 10.1155/2022/1250264

**Published:** 2022-12-31

**Authors:** Josiane Claire Sonkoue Lambou, Michel Noubom, Boris Emmanuel Djoumsie Gomseu, Wiliane Jean Takougoum Marbou, Jean-De-Dieu Tamokou, Donatien Gatsing

**Affiliations:** ^1^Research Unit of Microbiology and Antimicrobial Substances, Department of Biochemistry, Faculty of Science, University of Dschang, P. O. Box 67, Dschang, Cameroon; ^2^Departement of Microbiology, Hematology and Immunology, Faculty of Medicine and Pharmaceutical Sciences, University of Dschang, Dschang, Cameroon

## Abstract

Urinary tract infections (UTIs) in patients with diabetes are a major public health problem worldwide, particularly in developing countries. This study assessed the resistance profile of *Escherichia coli* and biochemical abnormalities in controlled and uncontrolled type 2 diabetic patients. A cross-sectional study was conducted at the Douala Laquintinie Hospital from January, 2020, to July, 2021, on the diabetic and nondiabetic participants. The clinical symptoms and biochemical parameters of patient having UTIs were measured using standard methods. *E. coli* was isolated from urine and an antibiotic susceptibility test was performed using the Kirby-Bauer Agar diffusion method. A total of 851 participants were included with a mean age of 48.54 years. Three hundred and forty-six (40.67%) were nondiabetic, 226 (26.56%) were diabetic patients with balanced blood sugar levels (i.e., glycosylated haemoglobin (HbA1c) is normal), and 279 (32.78%) were diabetic patients with unbalanced blood sugar levels (i.e., patients having an abnormal HbA1c). The prevalence of UTI caused by *E. coli* was significantly (*p* < 0.001) higher in diabetics with unbalanced blood sugar levels (15.41%) and diabetics with balanced blood sugar levels (9.73%) compared to nondiabetics (0.87%). Significant (*p* < 0.001) high frequencies of polyuria (48.39%), proteinuria (29.75%), leukocyturia (27.96%), and polyphagia (8.24%) were observed in diabetic participants with unbalanced blood sugar levels. Significantly (*p* < 0.001) high average values of aspartate transaminase (25.34; 27.07; 29.93), alanine transaminase (26.08; 27.38; 28.20), creatininemia (8.15; 9.67; 11.31), total cholesterol (1.57; 1.83; 2.63), and atherogenic index (3.81; 6.56; 11.73) were noted in nondiabetics, balanced, and unbalanced blood glucose diabetics, respectively. *E. coli* showed a high level of resistance to ciprofloxacin (30%), amoxicillin (10.8%), and ofloxacin (9.3%) in diabetic participants with unbalanced blood sugar levels. The antibiotic resistance patterns of the *E. coli* to triple, quadruple, and quintuple antibiotics were higher when participants had diabetes and even more when diabetes was not controlled. The present findings underline an increased susceptibility of diabetic patients with unbalanced blood sugar levels to multidrug resistant *E. coli*. Further studies should be conducted to determine the causal association between uncontrolled diabetes and bacterial multidrug resistance.

## 1. Introduction

Diabetes is a major public health problem in the world and particularly in developing countries where care and follow-up methods are less respected [1]. Type 2 diabetes is the main type of diabetes defined as high sugar in the blood resulting from a defect in insulin secretion and/or action [2]. Lack of production or inability to respond to insulin causes an increase in blood sugar, which in turn leads to a damage affecting several organs or systems, in particular the vessels and nerves [3]. Glycosylated haemoglobin (HbA1c) is a form of haemoglobin that nonenzymatically bound to sugar. Controlled diabetes is generally defined as patients whose HbA1c is normal while uncontrolled diabetes is defined as patients having an abnormal HbA1c [4, 5]. A high and uncontrolled blood glucose level has a damaging impact on several body organs functioning therefore leading to nephropathy, retinopathy, neuropathy, infarction, hypertension, arteriosclerosis, and stroke [6]. Controlled diabetes is associated with a significant decrease in the incidence of neuropathic and microvascular complications in type 2 diabetic patients [7, 8]. Diabetes is also often linked to other complications, such as sleep apnea, capsulitis, erectile dysfunction, yeast infections, periodontitis, and urinary tract infections [6]. The immune capacities in diabetics are depressed and therefore expose patients to urinary, skin, and pulmonary infections [9]. In fact, hyperglycemia was demonstrated to impair the chemotaxis of neutrophils, phagocytosis, superoxide production, IL-6 expression in intermediate monocyte, IL-17A expression, and bacterial activity [9]. These factors explain the increased frequency of urinary tract infections (UTIs) [9]. It has been demonstrated both in mice and humans that diabetics with good glycaemic control and healthy controls were less susceptible to bacterial infections [10].

UTIs are common in diabetics, sometimes severe due to the state of immunosuppression. The cytobacteriological examination of the urine is the key examination for the definitive UTIs diagnosis. Several bacteria such as *Klebsiella pneumoniae*, *Escherichia coli*, and *Acinetobacter baumanii* can cause urinary tract infections in diabetics [11]. *E. coli* is one of the most common pathogen in diabetic patients that can affect all organs and systems [12]. It was demonstrated that the administration of therapeutic insulin has an important role in the spread of infectious diseases in diabetic patients [13]. Indeed, the presence of insulin in blood increases the proliferation of many Enterobacteriaceae such as *Enterococcus faecalis* and *Klebsiella pneumoniae* [13]. Insulin stimulates the expression of the enzyme virulence factor aspartyl proteinase, which increases metabolic activity and biofilm formation leading to bacterial resistance [14]. The resistance of bacteria to antibiotics in the general population and in diabetics in particular is a public health problem. The continuous evolution of bacterial resistance requires a need of up-to-date knowledge of the rate of resistance as well as an understanding of the risk factors involved in this resistance. To the best of our knowledge, there are no studies involving infected diabetics with both balanced and unbalanced blood sugar levels in Cameroon. The present study aimed to assess the resistance profile of *E. coli* and biochemical abnormalities in diabetic patients with balanced and unbalanced blood glucose levels.

## 2. Methods

### 2.1. Study Design, Population, and Sampling Method

A cross-sectional hospital based study was carried out in the Laboratory of the Laquintinie Hospital from January 2020 to July 2021. Participants were diabetic and nondiabetic patients visiting Laquintinie Hospital diabetic unit for their regular follow-up (type 2 diabetic patients) and infectious diseases unit (nondiabetic) willing to participate in the study. Informed consents of all the participant were collected before sampling. Patients were recruited on the basis of the criteria listed in Table 1.

In this study, serum and urine samples were collected from patients with frequent/severe episodes of hypoglycaemia who agreed and provided their consent to participate in the study. Fasting blood sugar is blood glucose measured from venous blood after at least 8 hours of overnight fasting. Patients with balanced blood sugar levels or controlled diabetic patients were defined as patients whose glycosylated haemoglobin (HbA1c) was below 8% [15] and an average fasting blood sugar measurement of three consecutive visits was between 70 and 120 mg/dL. Patients with unbalanced blood sugar levels or uncontrolled diabetic patients were defined as patients having a glycosylated haemoglobin (HbA1c) above 8% and an average fasting blood sugar measurement for three consecutive visits above 120 or below 70 mg/dL [16]. Finally, 851 participants with 346 nondiabetics, 226 controlled diabetics, and 279 uncontrolled diabetics were included in the study.

### 2.2. Blood and Biochemical Measurements

The antecubital vein of the forearm was selected and disinfected with a cotton wool swab impregnated with 70% alcohol. Five millilitres of venous blood were collected into a dry tube (5 mL) and EDTA tube (5 mL) prelabelled with an anonymised patient code. The blood sample in the dry tube was allowed to clot completely before centrifugation at 3000 rpm for 15 min. Serum was separated from the clot into tightly screwed microfuge tubes and stored at −20°C. These frozen serums were later analysed for assessment of the biochemical parameters. Serum alanine aminotransferase (ALAT), serum aspartate aminotransferase (ASAT), fasting blood sugar, creatinine, uric acid, C-reactive protein (CRP), urea, sodium, potassium, chlorine, total cholesterol, HDL-C, LDL-C, and triglycerides (TG) were measured using the methods described in SPINREACT Commercial kits (SPINREACT, Spain). Glycated haemoglobin (HbA1C) test was performed using quantitative method of haemoglobin glycation [17].

### 2.3. Urine Collection

The first urine in the morning was collected. Sampling was conducted in a sterile jar and it was immediately inoculated on culture media. Identification of *E. coli* from positive culture plates was performed with the use of standard microbiology techniques, which included colony morphology, Gram stain, biochemical tests, and serotyping [18]. The Analytical Profile Index (API 20E) was used to support the bacterial identification process (BioMerieux, France).

### 2.4. Antibiotic Susceptibility Testing

*In vitro* susceptibility of *E. coli* isolates against antibiotics was performed using Kirby Bauer Agar Diffusion Method [19]. The antibiotics tested were penicillins (ampicillin, 10 *μ*g; ticarcillin, 75 *μ*g; piperacillin, 75 *μ*g; oxacillin, 1 *μ*g; and amoxicillin, 25 *μ*g), cephalosporins (ceftriaxone, 30 *μ*g; cefoxitin, 30 *μ*g; cefalotin, 30 *μ*g; cefepime, 30 *μ*g; cefixime, 5 *μ*g; cefotaxime, 30 *μ*g; and ceftazidime, 10 *μ*g), fluoroquinolones (ciprofloxacin, 5 *μ*g and ofloxacin, 5 *μ*g), glycopeptides (vancomycin, 5 *μ*g), lincosamides (clindamycin, 2 *μ*g), aminoglycosides (amikacin, 30 *μ*g and gentamicin, 30 *μ*g), and trimethoprim-sulfamethoxazole (25/23.75 *μ*g) (BIORAD, France). Each assay was performed in triplicate. Antibacterial activity of antibiotics against *E. coli* isolates was evaluated by measuring the clear zone of growth inhibition (diameter of zone of inhibition) on agar surface around the discs as per clinical and laboratory standards institute (CLSI) guideline [20]. *E. coli* classified as susceptible, intermediate, and resistant strains according to the criteria of the CLSI [20]. *Escherichia coli* isolates were regarded as multidrug resistant (MDR) when they were resistant to one or more antibiotics in three or more classes of antimicrobials that the isolate is expected to be susceptible.

### 2.5. Ethical Approval

The Regional Ethics Committee of Littoral, Cameroon (Ref no 2367CEI-UDo/07/2020/T) approved the experimental procedures and protocols used in this study. The concept of the study was explained to the participants and a signed informed consent to participate in the study was obtained. A designed questionnaire was administered to each participant to collect dietary habits, biological status, socio-economic, and demographic information.

### 2.6. Statistical Analysis

The Chi-square test was used to compare the frequencies of risk factors in the different groups in order to infer a relationship between these risk factors and the medical condition of the participants. The determined biochemical parameters were subjected to analysis of variance and when differences existed for each biochemical parameter taken individually, the Waller-Duncan test at a 5% probability threshold was used to separate these means. Pearson's bivariate correlation was used to determine the association between risk factors and patient biochemical parameters. The visual dashboard test made it possible to compare the odds ratios at 95% CI of the risk factors in different groups in order to infer a possible association between these risk factors and the medical condition of the participants. All of these analyses were performed using SPSS 26.0 software.

## 3. Results

A total of 851 volunteer's participants (average age 48.66 ± 13.33 years) of both gender, who attended the Laquintinie Hospital, were enrolled into the study. They consisted of 346 (40.65%) nondiabetics, 226 (26.55%) controlled diabetics, and 279 (32.78%) uncontrolled diabetics.

### 3.1. Baseline Characteristics of Study Participants

The study shows that nondiabetics and type 2 diabetic patients vary significantly with age groups (*p* < 0.001), sex (*p*=0.025), marital status (*p* < 0.001), level of education (*p* < 0.001), profession (*p* < 0.001), and body mass index (*p* < 0.001) (Table 2).

The highest frequencies (*p* < 0.001) of polyuria (48.39%) followed by leukocyturia (27.96%) and polyphagia (8.24%) were observed in unbalanced blood sugar diabetic patients (Table 2). We also observed the highest frequency of dysuria (9.73%) and proteinuria (34.07%) in balanced blood sugar diabetics patients. The different frequencies of all clinical symptoms showed significant differences between the three groups of patients (*p* < 0.001) (Table 3).

### 3.2. Prevalence of Urinary Tract Infections Caused by *E. coli* as a Function of Diabetic Status

**Figure 1:**
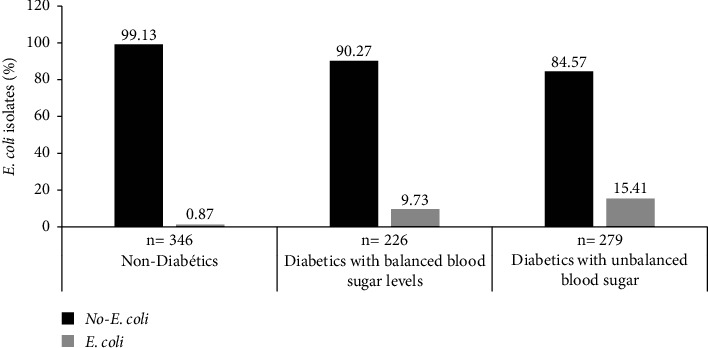
Distribution of *E. coli* isolates as a function of diabetic status.

The prevalence of urinary tract infections caused by *E. coli* was significantly (*p* < 0.001) higher in diabetics with unbalanced blood sugar levels (15.41%) and in diabetics with balanced blood sugar levels (9.73%) compared to that recorded in nondiabetics (0.87%) (Figure 1).

### 3.3. Biochemical Profiles as a Function of Diabetic Status

The average values of ASAT, ALAT, uremia, chloremia, uricemia, CRP, creatininemia, triglyceridemia, LDL, total cholesterol, atherogenic index, LDL/HDL index, fasting blood sugar, and glycated haemoglobin increased significantly (*p* < 0.001) with diabetes status whereas those of potassium, sodium, creatinine clearance, and HDL-cholesterol decreased significantly (*p* < 0.001) (Table 4). The mean values of LDL-cholesterol, atherogenic index, LDL/HDL index, CRP, sodium (in diabetic patients), HDL-cholesterol (in controlled diabetics), total cholesterol, HbA1C, and glycemia (in uncontrolled diabetics) are out of the normal ranges. However, the mean values of other biochemical parameters were within the normal ranges.

### 3.4. Biochemical Profiles of the Study Population According to *E. coli* Infection Status

*E. coli* infection rate was significantly higher in type 2 diabetic patients with unbalanced blood glucose levels (26.88%) compared with those recorded in nondiabetics (4.04%) and type 2 diabetic patients with balanced blood glucose levels (15.92%) (Table 5). The results also showed that the frequencies of hyper ASAT, hyper CRP, hypercreatininemia, hypercholesterolemia LDL, moderate renal failure, proteinuria, hypernatremia, hyperkaliemia, hypertriglyceridemia, and abnormal LDL-cholesterol/HDL-cholesterol index were significantly higher in *E. coli* infected diabetics with unbalanced blood sugar levels compared to noninfected diabetics with unbalanced blood sugar levels (Table 5).

### 3.5. *E. coli* Antibiotic Resistance Profile as a Function of Diabetic Status

**Figure 2:**
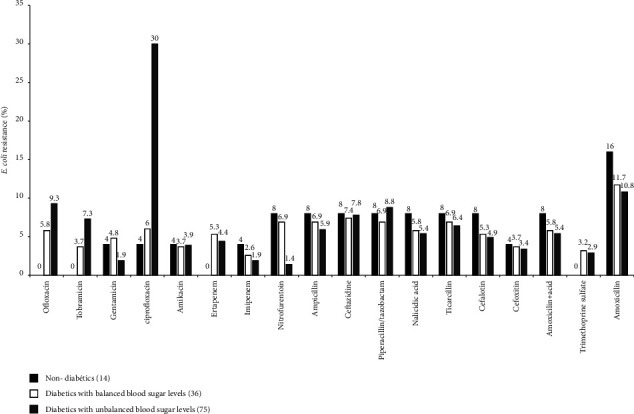
Distribution of *E. coli* antibiotic resistance profile as a function of diabetic status.

The resistance rates of *E. coli* to the commonly used antimicrobials are shown in Figure 2. *E coli*, as the most conducive pathogen of urinary tract infections, showed the highest level of resistance to ciprofloxacin (30%), amoxicillin (10.8%), and ofloxacin (9.3%) in type 2 diabetic patients with unbalanced blood sugar levels. In diabetic participants with balanced blood sugar levels, *E. coli* displayed resistance to amoxicillin (11.9%) whereas in nondiabetic participants, *E. coli* isolates were found to have high levels of resistance to amoxicillin (16%). Moreover, ofloxacin (0%), ertapenem (0%), tobramicin (0%), and trimethoprim sulfate were the most effective antimicrobial agents for *E. coli* infection in nondiabetic participants (Figure 2).

### 3.6. Multiple Antibiotics Resistance of *E. coli* Isolates as a Function of Diabetic Status

**Figure 3:**
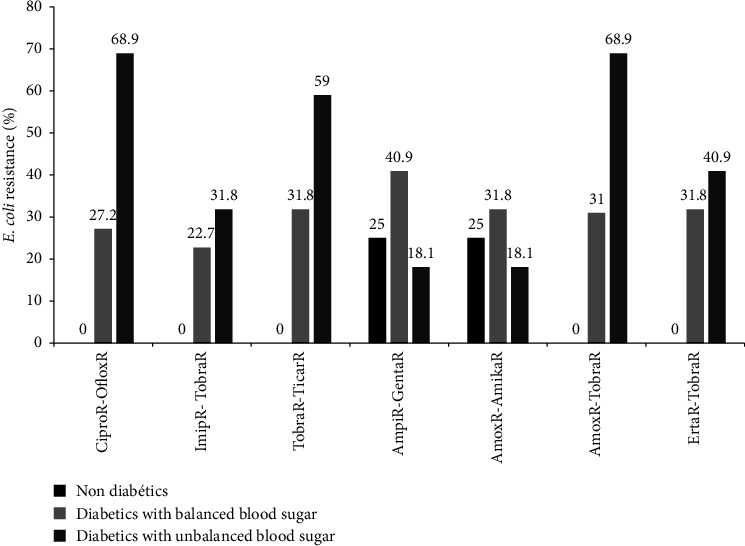
Distribution of *E. coli* resistance to double antibiotics as a function of diabetic status. Amika: amikacin, Amox: amoxicillin, Ampi: ampicillin, Cipro: ciprofloxacin, Genta: gentamicin, Oflox: ofloxacin, Erta: ertapenem, Ticar: ticarcillin, Tobra: tobramicin, Imip: imipenem, Nitro: nitrofurantoin, and R: resistant.

*E. coli* isolates showed the highest level of double antibiotics resistance to ciprofloxacin-ofloxacin (68.9%), amoxicillin-tobramicin (68.9%), and tobramycin-ticarcillin (59%) in type 2 diabetes with unbalanced blood sugar levels (Figure 3). In nondiabetic participants, *E. coli* was 100% sensitive to ciprofloxacin-ofloxacin, imipenem-tobramycin, tobramycin-ticarcillin, amoxicillin-tobramycin, and ertapenem-tobramicin (Figure 3).

**Figure 4:**
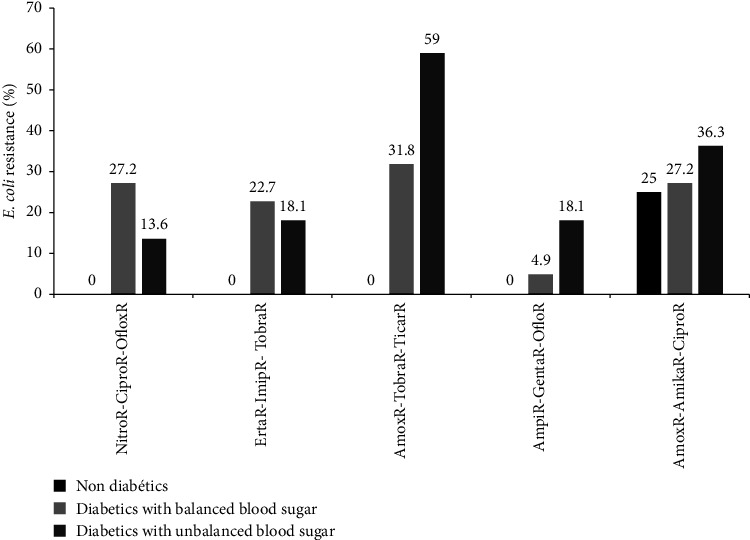
Distribution of *E. coli* resistance to triple antibiotics as a function of diabetic status. Amika: amikacin, Amox: amoxicillin, Ampi: ampicillin, Cipro: ciprofloxacin, Erta: ertapenem, Genta: gentamicin, Oflox: ofloxacin, Ticar: ticarcillin, Tobra: tobramicin, Imip: imipenem, and Nitro: nitrofurantoin.

The antibiotic resistance patterns of the *E. coli* to triple antibiotics are shown in Figure 4. *E. coli* isolates were resistant to amoxicillin-tobramycin-ticarcillin (59%) and to amoxicillin-amikacin-ciprofloxacin (36.3%) in type 2 diabetic patients with unbalanced blood sugar while in participants with balanced blood sugar, high resistance rate was observed only to amoxicillin-tobramycin-ticarcillin (31.8%). In non-diabetic participants, *E. coli* showed 100% susceptibility to nitrofurantoin-ciprofloxacin-ofloxacin, ertapenem-imipenem-tobramycin, amoxicillin-tobramycin-ticarcillin, and ampicillin-gentamycin-ofloxacin (Figure 4).

**Figure 5:**
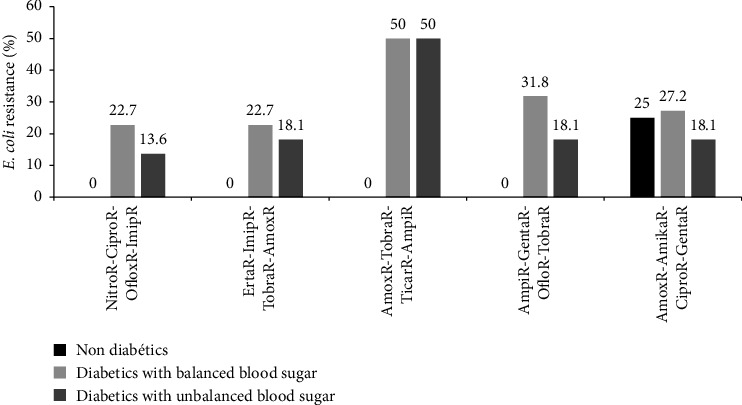
Distribution of *E. coli* resistance to quadruple antibiotics as a function of diabetic status. Amika: amikacin, Amox: amoxicillin, Ampi: ampicillin, Cipro: ciprofloxacin, Erta: ertapenem, Genta: gentamicin, Oflox: ofloxacin, Ticar: ticarcillin, Tobra: tobramicin, Imip: imipenem, and Nitro: nitrofurantoin.

The quadruple antibiotic resistance patterns of the *E. coli* are shown in Figure 5. *E. coli* displayed a resistance rate of 50% to amoxicillin-tobramycin-ticarcillin-ampicillin in type 2 diabetic patients with balanced and unbalanced blood sugar levels. However, in nondiabetes participants, *E. coli* was 100% sensitive to nitrofurantoin-ciprofloxacin-ofloxacin-imipenem, ertapenem-imipenem-tobramycin-amoxicillin, amoxicillin-tobramycin-ticarcillin-ampicillin, and ampicillin-gentamycin-ofloxacin-tobramycin (Figure 5).

**Figure 6:**
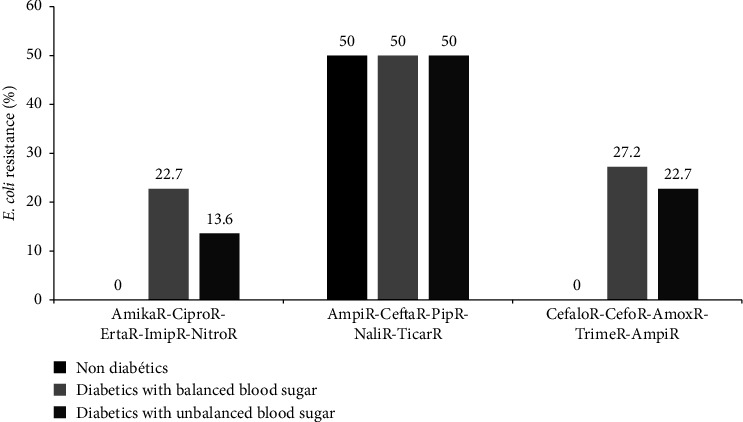
Distribution of *E. coli* resistance to combination of five commonly used antibiotics as a function of diabetic status. Amika: amikacin, Amox: amoxicillin, Ampi: ampicillin, Cefalo: cefalotin, Cefo: cefotaxime, Cefta: ceftazidime, Cipro: ciprofloxacin, Erta: ertapenem, Pip: piperacillin, Ticar: ticarcillin, Trime: trimethoprim-sulfamethoxazole, Imip: imipenem, Nitro: nitrofurantoin, and Nali: nalidixic acid.

The resistance rates of the isolated *E. coli* to the combination of five commonly used antibiotics are shown in Figure 6. *E. coli* was 50% resistant to ampicillin-ceftazidime-piperacillin-nalidixic acid-ticarcillin both in diabetics with balanced and unbalanced blood sugar levels. In nondiabetes participants, *E. coli* was 100% sensitive to amikacin-ciprofloxacin-ertapenem-imipenem-nitrofurantoin and cefalotin-cefoxitin-amoxicillin-trimethoprim-ampicillin (Figure 6).

## 4. Discussion

Urinary tract infections in patients with diabetes are a major public health problem worldwide [12]. In the present study, we assessed the resistance profile of *Escherichia coli* and biochemical abnormalities in type 2 diabetic patients with balanced and unbalanced blood glucose levels. The results of the current investigation showed that, urinary tract infections caused by *E. coli* vary significantly with age groups (*p* < 0.001), sex (*p*=0.025), marital status (*p* < 0.001), level of education (*p* < 0.001), and profession (*p* < 0.001). This finding was consistent with that of a study conducted in Mulago Hospital in Uganda [21].

The highest frequencies of polyuria (48.39%), leukocyturia (27.96%), and polyphagia (8.24%) were observed in unbalanced blood sugar diabetic patients (*p* < 0.001). Polyuria has been demonstrated as one of the most prevalent symptoms of diabetes [22]. Diabetic patients often present a variety of symptoms indicating urinary tract infections, including proteinuria and leukocyturia. This study also revealed the highest frequency of proteinuria and dysuria (9.73%) in diabetics with balanced blood sugar levels. The prevalence of urinary tract infection caused by *E. coli* was significantly (*p* < 0.001) higher in diabetics with unbalanced blood sugar levels (15.41%) and diabetics with balanced blood sugar levels (9.73%) compared to nondiabetics (0.87%). The reason behind the association between hyperglycemia and increased frequency of *E. coli* UTIs is an immunocompromised state of the diabetic patients. In fact, uncontrolled hyperglycemia alters the overall immunity of diabetic patients through various mechanistic pathways which make the diabetic patient an immunocompromised subject [23]. Other conditions related with type 2 diabetes such as obesity have been associated with an increased risk of infections due to the role of adipose tissue in the production of proinflammatory cytokines (tumor necrosis factor (TNF)-*α*, interleukin (IL) 6, IL-1*β*, IL-18, monocyte chemoattractant protein (MCP)-1), proinflammatory adipokines, and other inflammatory products [24, 25]. To the best of our knowledge, there are no studies involving infected diabetics with both balanced and unbalanced blood sugar levels in Cameroon.

The findings of the present investigation revealed that participants with balanced and unbalanced blood sugar levels exhibit significant heterogeneity in the mean values of ASAT, ALAT, CRP, uremia, chloremia, creatininemia, triglyceridemia, LDL-cholesterol, total cholesterol, atherogenic index, and glycated haemoglobin. Lipid profile abnormalities, renal biochemical disorders, and liver steatosis are known characteristics of diabetes [26, 27]. Hence, patients with diabetes mellitus and impairments of hepatic, renal, and heart functions experience lower clinical success rates than patients without these comorbidities and may also have a longer length of hospital stay and higher risk of adverse drug events.

In diabetic patients, cardiovascular diseases represent the main burden in terms of morbidity and mortality [28], whereas infections are increasing both in frequency and severity in these subjects [29]. In the current study, cardio-metabolic disorders (hyper CRP, hyper-LDL-cholesterolemia, hypertriglyceridemia, hyperkaliemia, and hypernatremia) and renal biochemical abnormality (hypercreatininemia, proteinuria) were significantly higher (*p* < 0.001) in *E. coli* infected diabetics with unbalanced blood sugar levels compared to noninfected diabetics with unbalanced blood sugar levels.

In fact, the presence of *E. coli* leads to an increase of CRP, creatininemia, glycemia, LDL cholesterolemia, triglyceridemia, hyperkaliemia, and hypernatremia, and this is more accentuated in diabetic patients with unbalanced glucose levels. The most elevated frequencies of hypercreatininemia and proteinuria in infected participants can be linked to the effect of *E. coli* toxins on the kidneys [30]. Hence, the increases of cardio-metabolic and renal biochemical parameters suggest the alterations of the kidney and heart functions in the uncontrolled diabetic patients. In addition to urinary tract infections (UTIs), diabetes has shown to increase the risk of severe Gram-positive infections, hospital-acquired postoperative infections, bacteraemia, tuberculosis, and pneumonia [31, 32].

Another interesting finding of this study was the highest level of *E. coli* resistance to ciprofloxacin (30%), amoxicillin (10.8%), and ofloxacin (9.3%) in diabetic patients with unbalanced blood sugar levels while in diabetic participants with balanced blood sugar levels, *E. coli* demonstrated resistance to amoxicillin (11.9%). Studies have shown that diabetic patients are prone to have various kinds of resistant bacteria than nondiabetic patients [33, 34]. This high incidence rate of antimicrobial resistance is attributed to the prescribing pattern of antibiotics for UTIs among diabetic and nondiabetic patients and the altered immune function in patients with diabetes mellitus caused by hyperglycemia [9]. Comorbid renal, hepatic, and heart impairments in patients with diabetes mellitus are associated with impairments in host defenses [31]. Although available guidelines [35] recognize type 2 diabetes as a risk factor for bacteriuria, little is said about antibiotic resistance or appropriate treatment choices for people with type 2 diabetes. Our work could be considered by these guidelines to suggest pragmatic approaches in people with type 2 diabetes. Thus, first, we could carefully adopt empiric treatment considering that the choices for the general population may not be ideal for people with type 2 diabetes. Second, an antibiogram before starting any empiric antibiotic therapy is needed. Finally, we recommend a map of the antibiotic resistance profile in people with type 2 diabetes and urinary tract infections. This evidence would have a positive impact on guiding empirical treatment for people with type 2 diabetes.

The result also showed a strong resistance of *E. coli* isolated from uncontrolled diabetics to antibiotics of quinolone and Beta-lactam families. Hence, clinicians should be vigilant in recognizing the potential for infection with multidrug-resistant (MDR) organisms, especially MDR *E. coli* in type 2 diabetics and initiating therapy with appropriate antibiotics. In recent studies, it has been shown that antimicrobial resistance to quinolones and Beta-lactams is rising worldwide, especially in *E. coli* causing various infections [34, 36].

The study also revealed that the antibiotic resistance patterns of the *E. coli* to triple, quadruple, and quintuple antibiotics were highest when participants had type 2 diabetes and even more when diabetes was not controlled. This shows an increased susceptibility of uncontrolled diabetics to MDR *E. coli*. Immunocompromised state of uncontrolled diabetics and frequent antibiotic use may be associated with antibiotic resistance of *E. coli* observed in this study. In addition, the pharmacokinetics of antibiotics in obese diabetics can lead to suboptimal levels of antibiotic concentrations and increase the risk for antibiotic resistance [37]. The above results stressed that MDR with first-line antibiotics in patients with type 2 diabetes would impose a heavy economic burden. These patients may need a second appointment with their doctor and start a different antibiotic treatment. In the worst case, the infection could progress and develop complications. The findings of this study indicated that hyperglycemia played a much more important role in *E. coli* distribution and antibacterial drug resistance. Further studies should be conducted to determine the causal association between uncontrolled diabetes and bacterial multidrug resistance.

The importance of this work can be attributed to the fact that, epidemiological data were obtained and this further helps the public health sector in the implementation of public health policies. Nevertheless, the cross-sectional nature coupled with certain uropathogenic bacteria that were not evaluated prevented it to be generalized, thus limiting this work.

## 5. Conclusion

This study aimed at assessing the resistance profile of *Escherichia coli* and biochemical abnormalities in diabetic patients with balanced and unbalanced blood glucose levels. The prevalence of urinary tract infection cause by *E. coli* was significantly higher in diabetics with unbalanced blood sugar levels and diabetics with balanced blood sugar levels compared to nondiabetics. Participants with balanced and unbalanced blood sugar levels exhibited significant heterogeneity in the mean values of biochemical parameters. The study revealed an increased susceptibility of diabetic patients with unbalanced blood sugar levels to multidrug resistant *E. coli*. These different observations therefore imply that the variation of physiological (blood glucose concentration) conditions can greatly influence the development of resistance and multidrug resistance. Further studies should be conducted to determine the causal association between uncontrolled diabetes and bacterial multidrug resistance.

## Figures and Tables

**Table 1:** Summary of study participant recruitment criteria.

Inclusion criteria^a^ (*n* = 890)	Noninclusion criteria^b^	Exclusion criteria^c^ (*n* = 39)
(i) Diabetics or nondiabetics at least (ii) 21 years old and giving a favorable opinion for free participation in the study	(i) Pregnant subjects (ii) Patients on antibiotic therapy (iii) Subjects having a prehistory of specific disorders such as liver diseases, malaria, cardiovascular diseases, and kidney failure (iv) Subjects who refused to sign the informed consent form	(i) Subjects with hemolyzed blood serum (ii) Subjects who did not provide the full amount of information required

^a^Inclusion criteria are characteristics that the prospective subjects must have if they are to be included in the study. ^b^Noninclusion criteria are characteristics that subjects must not have if they are to be included in the study. ^c^Excluded criteria are features that exclude subjects from the study in progress.

**Table 2:** Baseline characteristics of study participants.

Sociodemographic characteristics		Nondiabetics 346 (%)	Diabetics with balanced blood sugar levels 226 (%)	Diabetics with unbalanced blood sugar 279 (%)	*χ * ^2^	Confidence interval (*p* value)
*Age groups (years)*	(20–30)	61 (17.63)	5 (2.21)	3 (1.07)	166.46	0.62–0.89 (<0.001)
	(30–40)	127 (36.70)	41 (18.14)	43 (15.41)		
	(40–50)	68 (19.65)	53 (23.45)	77 (27.59)		
	(50–60)	44 (12.71)	75 (33.18)	82 (29.39)		
	(60–70)	26 (7.51)	42 (18.58)	65 (23.29)		
	(70–80)	18 (5.20)	6 (2.65)	8 (2.86)		
	(80–90)	2 (0.58)	4 (1.77)	1 (0.35)		

*Sex*	Male	117 (33.81)	73 (32.30)	119 (42.65)	7.35	0.91–1.03 (0.025)
	Female	229 (66.18)	153 (67.70)	160 (57.35)		

*Marital status*	Single	72 (20.81)	7 (3.10)	22 (7.89)	50.23	0.71–0.95 (<0.001)
	Married	241 (69.65)	198 (87.61)	224 (80.29)		
	Divorced	5 (1.45)	2 (0.88)	2 (0.72)		
	Widower	28 (8.09)	19 (8.41)	31 (11.11)		

*Level of education*	Illiterate	15 (4.34)	8 (3.54)	1 (0.36)	123.01	1.16–1.31 (<0.001)
	Primary	107 (30.92)	60 (26.55)	88 (31.54)		
	Secondary	133 (38.44)	155 (69.58)	175 (62.72)		
	University	91 (26.30)	3 (1.33)	15 (5.38)		

*Profession*	None	2 (0.58)	6 (2.65)	11 (3.94)	73.20	0.05–0.07 (<0.001)
	Student	30 (8.67)	2 (0.88)	5 (1.79)		
	Civil servant	73 (21.10)	35 (15.49)	21 (7.53)		
	Informal sector	146 (42.20)	99 (43.81)	117 (41.94)		
	Retirement	37 (10.69)	16 (7.08)	36 (12.90)		
	Household	58 (16.76)	68 (30.09)	89 (31.90)		

*Body mass index (BMI)*	Mean (kg/m^2^)	26.80 ± 0.17^a^	26.97 ± 0.36^a^	27.28 ± 0.24^a^		
	Loss weight (16 ≤ BMI ≤ 18.5)	3 (0.57)	6 (2.65)	2 (0.71)	109.15	0.66–0.84 (<0.001)
	Normal (18.5 < BMI ≤ 25)	100 (28.90)	87 (38.49)	101 (36.20)		
	Overweight (25 < BMI ≤ 30)	211 (60.98)	68 (30.08)	88 (31.54)		
	Moderate obesity (30 < BMI ≤ 35)	32 (9.35)	50 (22.12)	58 (20.78)		
	Severe obesity (35 < BMI ≤ 40)	0 (0)	14 (6.19)	22 (7.88)		
	Morbid obesity (BMI > 40)	0 (0)	1 (0.44)	8 (2.86)		

**Table 3:** Clinical features of the study participants.

Clinical symptoms		Nondiabetics 346 (%)	Diabetics with balanced blood sugar levels 226 (%)	Diabetics with unbalanced blood sugar 279 (%)	*χ * ^2^	Confidence interval (*p* value)
*Asthenia*	No	346 (100)	223 (98.67)	272 (97.49)	8.43	0.07–0.12 (<0.001)
	Yes	0 (00)	3 (1.33)	7 (2.51)		

*Vaginal itching*	No	345 (99.71)	220 (97.35)	276 (98.92)	6.62	0.09–0.18 (0.036)
	Yes	1 (0.29)	6 (2.65)	3 (1.08)		

*Dysuria*	No	346 (100)	204 (90.27)	260 (93.19)	31.84	0.31–0.60 (<0.001)
	Yes	0 (00)	22 (9.73)	19 (6.82)		

*Leukocyturia*	No	335 (97.10)	184 (81.42)	201 (72.04)	77.33	1.04–1.32 (<0.001)
	Yes	10 (2.90)	42 (18.58)	78 (27.96)		

*Lack of appetite*	No	346 (100)	226 (100)	278 (99.64)	2.05	0.58–0.68 (0.358)
	Yes	0 (00)	0 (00)	1 (0.36)		

*Diabetic wound*	No	346 (100)	174 (76.99)	211 (75.63)	92.94	1.00–1.24 (<0.001)
	Yes	0 (00)	52 (23.01)	68 (24.37)		

*Polakiuria*	No	346 (100)	225 (99.56)	279 (100)	2.76	0.25–0.27 (0.250)
	Yes	0 (00)	1 (0.44)	0 (00)		

*Polydipsia*	No	346 (100)	224 (99.12)	277 (99.53)	2.82	0.22–0.24 (0.243)
	Yes	0 (00)	2 (0.88)	2 (0.72)		

*Polyphagia*	No	346 (100)	216 (95.58)	256 (91.76)	28.40	0.26–0.35 (<0.001)
	Yes	0 (00)	10 (4.42)	23 (8.24)		

*Polyuria*	No	343 (99.13)	150 (66.37)	144 (51.61)	197.05	0.34–0.36 (<0.001)
	Yes	3 (0.87)	76 (33.63)	135 (48.39)		

*Proteinuria*	No	301 (86.99)	149 (65.93)	196 (70.25)	40.44	0.41–0.53 (<0.001)
	Yes	45 (13.01)	77 (34.07)	83 (29.75)		

**Table 4:** Mean values of biochemical parameters as function of diabetic status.

Biochemical parameters (normal range)	Nondiabetics	Diabetics with balanced blood glucose levels	Diabetics with unbalanced blood glucose levels	Total diabetics	*p* value
ASAT (10–40 IU)	25.34 ± 0.37	27.07 ± 0.56	29.93 ± 0.83	28.65 ± 0.53	0.005
ALAT (7–37 IU)	26.08 ± 0.45	27.38 ± 0.72	28.20 ± 0.78	27.82 ± 0.54	0.016
Creatininemia (0.6–1.3 g/l)	0.81 ± 0.01	0.96 ± 0.02	1.13 ± 0.25	20.98 ± 0.44	<0.001
Creatinine clearance (90–120 ml/min)	128.33 ± 2.80	88.38 ± 2.39	80.02 ± 2.08	10.55 ± 0.17	<0.001
Triglyceridemia (<1.35 g/l)	0.88 + 0.02	1.42 ± 0.51	1.79 ± 0.07	1.62 ± 0.05	<0.001
Urea (0.1–0.5 g/l)	0.27 ± 0.01	0.36 ± 0.10	0.38 ± 0.04	0.34 ± 0.02	0.012
LDL- cholesterol (<1.30 g/l)	0.60 ± 0.12	1.63 ± 0.2	2.12 ± 0.24	1.69 ± 0.06	<0.001
Artherogenicity index (≤4.5)	3.81 ± 0.47	5.84 ± 0.44	11.73 ± 1.32	9.09 ± 0.76	<0.001
LDL/HDL index (≤3.6)	1.27 ± 0.19	4.98 ± 0.36	9.78 ± 1.09	7.59 ± 0.63	<0.001
HDL cholesterol (≥0.4 g/l)	0.45 ± 0.00	0.37 ± 0.01	0.41 ± 0.02	0.39 ± 0.01	<0.001
Total cholesterolemia (<2.2 g/l)	1.57 ± 0.18	1.71 ± 0.06	2.63 ± 0.14	2.22 ± 0.09	<0.001
HbA1C (<8%)	3.51 ± 0.05	6.87 ± 0.06	9.89 ± 0.10	27.28 ± 0.24	<0.001
CRP (<6 mg/l)	10.30 ± 1.05	16.755 ± 1.56	23.76 ± 1.76	20.62 ± 1.21	<0.001
Na^+^ (135–145 meq)	142.78 ± 0.42	139.97 ± 0.36	139.7 ± 0.41	139.82 ± 0.28	<0.001
Cl^−^ (96–106 meq)	100.01 ± 0.41	107.41 ± 1	108.07 ± 0.95	107.82 ± 0.69	<0.001
K^+^ (3.5–5 meq)	4.17 ± 0.4	3.92 ± 0.13	3.67 ± 0.43	3.72 ± 0.31	<0.001
Uricemia (36–82 mg/dl)	45.84 ± 0.47	47.51 ± 0.99	52.74 ± 0.99	50.40 ± 0.54	<0.001
Glycemia (0.7–1.2 g/l)	0.90 ± 0.005	1.11 ± 0.006	2.44 ± 0.04	1.83 ± 0.04	<0.001

ALAT: alanine aminotransferase; ASAT: aspartate aminotransferase; CRP: C-reactive protein; LDL: low-density lipoproteins; HDL: high-density lipoproteins; HbA1C: glycated haemoglobin.

**Table 5:** Biochemical abnormalities as function of diabetic status and *E. coli* infection.

Biochemical parameters		*Nondiabetics 346 (%)*		*Diabetics with balanced blood glucose 226 (%)*		*Diabetics with unbalanced blood glucose levels 279 (%)*		*p* value
		Yes 14 (4.04%)	No 332 (95.95%)	Yes 36 (15.92%)	No 190 (84.07%)	Yes 75 (26.88%)	No 204 (73.11%)	
*Escherichia coli*								
HbA1C	HbA1C abnormal	0 (0%)	0 (0%)	0 (0%)	0 (0%)	75 (100%)	204 (100%)	<0.001
HDL	Hypocholesterolemia HDL	4 (28.57%)	49 (14.75%)	24 (66.66%)	128 (67.36%)	43 (57.33%)	139 (68.13%)	<0.001
Artherogenicity index total cholesterol/HDL-cholesterol	Double risk	5 (35.71%)	30 (9.03%)	23 (63.88%)	90 (47.36%)	45 (60%)	135 (66.17%)	<0.001
LDL	Hypercholesterolemia LDL	0 (0%)	4 (1.20%)	8 (22.22%)	72 (37.89%)	51 (68%)	103 (50.49%)	0.001
Triglyceride	Hypertriglyceridemia	0 (0%)	41 (12.34%)	18 (50%)	69 (36.31%)	51 (68%)	105 (51.47%)	0.001
Total cholesterol	Total hypercholesterolemia	0 (0%)	2 (0.60%)	7 (19.44%)	30 (15.78%)	32 (42.66%)	92 (45.04%)	0.01
Urea	Hyperuremia	0 (0%)	18 (5.42%)	12 (33.33%)	25 (13.15%)	15 (20%)	17 (8.33%)	0.02
Creatinine clearance	Moderate renal failure	3 (21.42%)	10 (3.01%)	5 (13.88%)	30 (15.78%)	27 (36%)	47 (23.03%)	0.001
	End-stage renal failure	0 (0%)	0 (0%)	0 (0%)	0 (0%)	0 (0%)	1 (0.49%)	
Creatinine	Hypercreatininemia	0 (0%)	1 (0.30%)	1 (2.77%)	16 (8.42%)	26 (34.66%)	33 (16.17%)	0.002
	Hypocreatininemia	0 (0%)	9 (2.71%)	0 (0%)	1 (0.52%)	0 (0%)	3 (1.47%)	
ALAT	Hyper ALAT	1 (7.14%)	20 (6.02%)	2 (5.55%)	25 (13.15%)	10 (13.33%)	26 (12.74%)	0.030
	Hypo ALAT	0 (0%)	5 (1.5%)	1 (2.77%)	0 (0%)	0 (0%)	0 (0%)	
ASAT	Hyper ASAT	0 (0%)	1 (0.30%)	2 (5.55%)	6 (3.15%)	11 (14.66%)	13 (6.37%)	<0.001
	Hypo ASAT	0 (0%)	3 (0.90%)	1 (2.77%)	0 (0%)	0 (0%)	0 (0%)	
Glycemia	Hyperglycemia	0 (0%)	0 (0%)	0 (0%)	0 (0%)	75 (100%)	204 (100%)	<0.001
Proteinuria	Positive	6 (42.85%)	39 (11.74%)	22 (61.11%)	55 (28.94%)	41 (54.66%)	42 (20.58%)	0.73
Uricemia	Hypouremia	1 (7.14%)	37 (11.14%)	10 (27.77%)	62 (32.63%)	15 (20%)	40 (19.6%)	0.002
K^+^	Hyperkaliemia	4 (28.57%)	59 (17.77%)	7 (19.44%)	6 (3.15%)	5 (6.66%)	7 (3.43%)	0.94
	Hypokaliemia	0 (0%)	60 (18.07%)	13 (36.11%)	53 (27.89%)	20 (26.66%)	62 (30.39%)	
CL^−^	Hyperchloremia	0 (0%)	9 (2.71%)	16 (44.44%)	76 (40%)	29 (38.66%)	74 (36.27%)	0.003
	Hypochloremia	0 (0%)	3 (0.90%)	0 (0%)	7 (3.68%)	5 (6.66%)	6 (2.94%)	
Na^+^	Hypernatremia	5 (35.71%)	126 (37.95%)	18 (50%)	32 (16.84%)	14 (18.66%)	28 (13.72%)	0.01
	Hyponatremia	0 (0%)	8 (2.40%)	0 (0%)	9 (4.73%)	6 (8%)	7 (3.43%)	
CRP	Hyper CRP	14 (21.42%)	50 (15.06%)	34 (94.44%)	49 (25.78%)	63 (84%)	83 (40.68%)	0.003
LDL-cholesterol/HDL-cholesterol	Abnormal	0 (0%)	5 (1.50%)	17 (47.22%)	95 (50%)	46 (61.33%)	102 (50%)	0.002

ALAT: alanine aminotransferase; ASAT: aspartate aminotransferase; CRP: C-reactive protein; LDL: low-density lipoproteins; HDL: high-density lipoproteins; HbA1C: glycated haemoglobin.

## Data Availability

The data sets used to support the findings of this study are available from the corresponding author upon request.
